# Untersuchungen zum Einfluss verschiedener Mund-Nase-Schutzmasken auf die beweissichere Atemalkoholmessung

**DOI:** 10.1007/s00194-022-00574-0

**Published:** 2022-05-16

**Authors:** J. Seibt, S. Heide, D. Budde, J. Pietsch

**Affiliations:** 1 Medizinische Fakultät Carl Gustav Carus, Institut für Rechtsmedizin, Technische Universität Dresden, Fetscherstr. 74, 01309 Dresden, Deutschland; 2 Dräger Safety AG & Co. KGaA, Lübeck, Deutschland

**Keywords:** Atemalkoholanalyse, FFP2-Maske, Op.-Maske, Maskenpflicht, Messeinfluss, Breath alcohol analysis, FFP2 mask, Surgical mask, Mandatory mask, Measurement influence

## Abstract

Anlässlich eines richterlichen Gutachtenauftrages zur Frage, ob das Tragen von Mund-Nase-Schutzmasken zu einer Verfälschung des Atemalkoholmesswertes zuungunsten des Angeklagten führen kann, erfolgte unter Pandemiebedingungen eine experimentelle Testreihe an 6 gesunden Proband*innen (4 Männer, 2 Frauen), die risikoarmen Freizeitalkoholkonsum betrieben. Pro Untersuchungstag wurde jeweils ein Maskentyp (Op.-Maske, Textilmaske, FFP2-Maske) untersucht. Nach Aufnahme einer individuell berechneten Menge Alkohol und einer 30-minütigen Resorptionsphase erfolgten in halbstündigen Abständen 6 aufeinanderfolgende Atemalkoholmessungen, wobei zwischen den Messungen jeweils im Wechsel eine der Masken oder keine Maske getragen wurde. Anschließend wurden Wertepaare für Zeiträume mit und ohne Maske gebildet und die stündlichen Atemalkoholabbauraten berechnet. Im Ergebnis unterschieden sich die Atemalkoholabbauraten mit und ohne Masken nicht voneinander. Fehlermeldungen durch das Atemalkoholmessgerät, die auf das vorherige Tragen einer Maske zurückzuführen sind, traten nicht auf.

## Abstract (en)

On the occasion of a judicial expert opinion on the question of whether wearing a face mask can lead to a falsification of the breath alcohol value leading to detriment of the accused, an experimental series of tests was carried out under pandemic conditions on six healthy test subjects (four men, two women), who practiced low-risk recreational alcohol consumption. On each day of the study one specific mask type (surgical mask, textile mask, FFP2 mask) was examined. After ingestion of an individually calculated amount of alcohol and a 30-min absorption phase, 6 consecutive breath alcohol measurements were carried out at 30-min intervals, with one mask or no mask worn between measurements. Subsequently, pairs of values for periods with and without a mask were formed and the hourly breath alcohol elimination rates were calculated. As a result, the breath alcohol elimination rates with and without masks did not differ from each other. There were no error messages from the breathalyzer that can be attributed to the previous wearing of a mask.

Im Zuge der pandemischen Lage in den Jahren 2020/2021 ist das Tragen von Mund-Nase-Schutzmasken (MNS) zur Eindämmung des Sars-CoV-2-Infektionsgeschehens mittlerweile zum Alltag geworden. Während von der Bevölkerung zu Beginn der Pandemie mangels Alternativen oft selbstgenähte Textilmasken aus Baumwollstoffen („community masks“) genutzt wurden, trat im Verlauf aufgrund strengerer Vorgaben im Infektionsschutzgesetz die Nutzung von medizinischen Masken und Atemschutzmasken der Kategorie 2 (FFP2/KN95) in den Vordergrund [21]. Eine im Zusammenhang mit der erweiterten Maskenpflicht erstmals aufgetretene Einlassung im Rahmen einer Ordnungswidrigkeit, dass das Tragen einer MNS vor der beweissicheren Atemalkoholmessung durch Ethanolretention in der Ausatemluft unter der Maske zu überhöhten Messwerten führen würde, sollte durch ein rechtsmedizinisches Gutachten überprüft werden.

## Einleitung

Das korrekte Tragen einer MNS führt zu einer spürbaren Erwärmung und Befeuchtung der Luft hinter der Maske und kann zu erhöhter Atemanstrengung mit Anstieg der Atem- und Herzfrequenz und damit einhergehendem Gefühl der Atemnot, Kopfschmerzen, Hitzegefühl oder Schwindel führen [6]. Im Zusammenhang mit der beweissicheren Atemalkoholmessung können insbesondere Änderungen der Atemtemperatur (z. B. durch Modulation der Atemtechnik) bekanntlich zu Messabweichungen führen, welche durch das Atemalkoholmessgerät nur bedingt detektiert werden können [18]. Im Rahmen einer kleineren experimentellen Testreihe konnte zudem bereits 2004 der Nachweis einer gewissen Akkumulation von CO_2_ unter OP-Masken durch Rückatmung erbracht werden, was zu einer messbaren, aber klinisch nichtrelevanten Erhöhung des CO_2_-Partialdrucks im Blut führte [3]. In späteren Untersuchungen konnte gezeigt werden, dass der CO_2_-Anstieg im Blut auch vom Maskentyp abhängig ist [6]. Ob das Tragen von MNS auch zu einer Verfälschung von Atemalkoholmesswerten führen kann, ist bislang nicht untersucht worden. Daher wurde am Institut für Rechtsmedizin Dresden eine praktisch-experimentelle Untersuchung initiiert, mit dem Ziel zu prüfen, ob das Tragen einer MNS vor der Atemprobenabgabe zu einer Fehlermeldung durch das Analysegerät führt, und ob es zu signifikanten Unterschieden zwischen den Alkoholabbauraten mit und ohne vorheriges Tragen einer MNS kommt. Die Studie erhielt durch die Ethikkommission der Medizinischen Fakultät Carl Gustav Carus der Technischen Universität Dresden ein positives Votum (BO-EK-523112020).

## Methode

Es wurden gesunde freiwillige Probanden (♂ *n* = 4, 28 bis 37 Jahre) und Probandinnen (♀ *n* = 2, 31 und 34 Jahre) aus dem Institut rekrutiert, die nach eigenen Angaben in ihrer Freizeit risikoarmen Alkoholkonsum genäß der Definition des Bundesministeriums für Gesundheit [2] betrieben (Tab. 1).

**Table 1:** 

Proband	Alter (in Jahren)	Geschlecht	BMI (kg/m^2^)	Aufgenommene Menge Alkohol (40 Vol.-%, in ml)^a^	Individueller Reduktionsfaktor r_i_	Anzahl der gültigen Messungen
A	34	Weiblich	19,5	128/170	0,71	17
B	33	Männlich	24,9	183/210	0,74	18
C	31	Weiblich	17,6	122/160	0,74	18
D	29	Männlich	29,7	210/260	0,64	18
E	37	Männlich	24,2	207/260	0,72	17
F	28	Männlich	24,4	198/250	0,72	19

^a^Tag 1/Tage 2 und 3

Nach Aufklärung, Einholung anamnestischer Angaben, ärztlicher Untersuchung und Ausschluss einer Schwangerschaft bei den Teilnehmerinnen erfolgte nach Berechnung der persönlichen Trinkmenge anhand der Widmark-Formel die kontrollierte Aufnahme eines hochprozentigen alkoholischen Getränks nach Wahl (Gin oder Rum, 40 Vol.-%; Tab. 1), um zu Versuchsbeginn eine theoretische Blutalkoholkonzentration von ca. 1,0 ‰ zu erreichen. Da bei der Alkoholmengenberechnung der individuelle Reduktionsfaktor r_i_ Anwendung fand, wurde kein zusätzliches Resorptionsdefizit berücksichtigt [1, 20]. Nach Konsum der bereitgestellten Menge Alkohol und anschließender 30-minütiger Resorptionsphase, in der weder gegessen noch geraucht oder getrunken werden durfte, erfolgten insgesamt 6 Messungen in halbstündlichen Abständen am Messgerät Dräger Alcotest 9510 DE (Fa. Dräger, Lübeck, Deutschland), jeweils im Wechsel mit und ohne vorherigem Tragen einer MNS (Tab. 2).

**Table 2:** 

Zeit	Versuchsablauf
Vor 08:00 Uhr	Frühstück
08:30–9:30 Uhr	Trinkzeit
09:30–10:00 Uhr	Resorptionsphase (ohne MNS)
10:00–10:10 Uhr	1. AAK-Messung (1/7/13)
10:10–10:30 Uhr	Kontinuierliches Tragen des MNS
10:30–10:40 Uhr	2. AAK-Messung (2/8/14)
10:40–11:00 Uhr	Wartezeit ohne MNS
11:00–11:10 Uhr	3. AAK-Messung (3/9/15)
11:10–11:30 Uhr	Wartezeit mit MNS
11:30–11:40 Uhr	4. AAK-Messung (4/10/16)
11:40–12:00 Uhr	Wartezeit ohne MNS
12:00–12:10 Uhr	5. AAK-Messung (5/11/17)
12:10–12:30 Uhr	Wartezeit mit MNS
12:30–12:40 Uhr	6. AAK-Messung (6/12/18)
12:40–15:30 Uhr	Ausnüchterungsphase

*AAK* Atemalkoholkonzentration, *MNS* Mund-Nase-Schutzbedeckung

**Figure 1:**
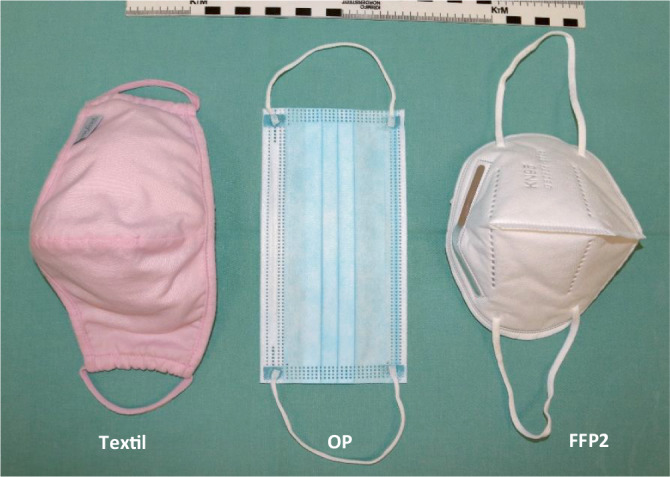

An den 3 nicht direkt aufeinanderfolgenden Untersuchungstagen wurde jeweils ein Maskentyp (Abb. 1) getestet. Dies waren industriell gefertigte, wiederverwendbare Textilmasken (Fa. Van Laack, Baumwolle/Polyester, dreilagig, Einheitsgröße für Erwachsene), medizinische Einweg-OP-Masken (Fa. Zhende, Vliesstoff, dreilagig, Einheitsgröße mit elastischen Ohrschlaufen) und FFP2-Atemschutzmasken (Fa. Zhejiang Xichen Medical Technology Co. Ltd, vierlagig, KN95 mit elastischen Ohrschlaufen und Metallnasenbügel).

**Figure 2:**
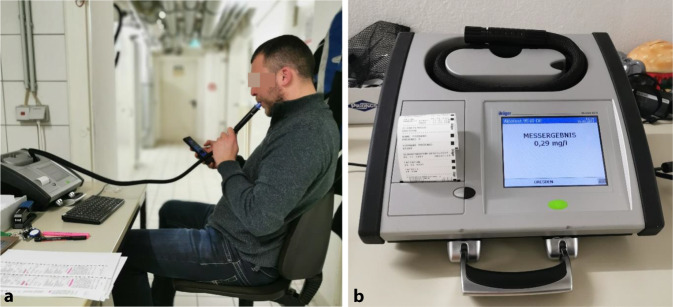

Die Atemalkoholmessungen (Abb. 2a) wurden mit dem Messgerät Dräger Alcotest 9510 DE (Seriennummer ARDE-0002) der Fa. Dräger, Lübeck (Abb. 2b) durchgeführt, welches direkt vom Hersteller zur Verfügung gestellt wurde. Es handelte sich um ein gerade instandgesetztes und justiertes Schulungsgerät. Ein Messvorgang bestand nach automatisierter interner Gerätekalibrierung aus 2 Einzelmessungen, wobei die erste Ethanolbestimmung in der Ausatemluft durch ein elektrochemisches, die zweite durch ein infrarotoptisches Messverfahren erfolgte. Anschließend wurde aus den Ergebnissen der Einzelmessungen (mit 3 Nachkommastellen) der Mittelwert gebildet, welcher durch das Gerät als auf 2 Nachkommastellen verkürztes Endergebnis in Milligramm pro Liter (mg/l) ausgegeben wurde. Traten während des Messvorgangs Abweichungen auf, wurde der Messvorgang vom Gerät abgebrochen und kein gültiges Ergebnis ausgegeben. Jeder Messvorgang, einschließlich evtl. aufgetretener Fehler oder unzulässiger Abweichungen, wurde im ausgegebenen Papierausdruck protokolliert. Ein komplikationsfreier Messvorgang mit 2 Atemprobenabgaben dauerte ca. 5 min [5].

Die Standardmessabweichung des Geräts liegt für Messwerte zwischen 0 und 0,400 mg/l bei < 0,006 mg/l, im Messbereich > 0,400–1,000 mg/l bei < 1,5 % vom Messwert. Messergebnisse unter 0,05 mg/l werden als 0,00 mg ausgegeben [5].

Die Teilnehmer*innen wurden angehalten, die Masken möglichst eng am Gesicht zu tragen, um Randleckagen weitestgehend zu unterbinden. Um die Untersuchung so realitätsnah wie möglich entsprechend den Abläufen bei der polizeilichen Messung zu gestalten, wurden die Probanden und Probandinnen bei den Messungen nach vorherigem Tragen der MNS aufgefordert, direkt vor der Atemprobenabgabe tief zu inspirieren und erst dann die Maske herunterzuziehen, um in den Schlauch zu atmen. In den 2–3 min zwischen den beiden Einzelmessungen wurde die Maske nicht wieder aufgesetzt. Weiterhin wurden die Teilnehmer*innen aus Gründen der Vergleichbarkeit gebeten, an den Untersuchungstagen das Gleiche zu frühstücken, um ähnliche Ausgangsbedingungen zu schaffen. Zwischen den Messungen war Nahrungsaufnahme, Kaugummikauen, Bonbonluschen und Rauchen verboten. Nach Beendigung der Messserien erfolgte die Ausnüchterung unter ärztlicher Aufsicht.

Analog zu vorangegangenen AAK-Studien im Institut [14] erfolgte die Auswertung nicht mit dem auf 2 Dezimalstellen verkürzten Ausgabewert des Gerätes, sondern mit dem Mittelwert der beiden Einzelmessungen (3 Nachkommastellen), was die Messwertpräzision zwar nicht erhöhte, wodurch aber auch detailliertere Unterschiede zwischen den Messwerten sichtbar wurden. Die erhobenen Daten wurden deskriptiv statistisch ausgewertet. Anschließend wurden Wertepaare zwischen 2 aufeinanderfolgenden Messungen gebildet. Im Zeitraum zwischen diesen Messungen wurde entweder kontinuierlich eine der 3 MNS oder keine MNS getragen. Für die erstellten Wertepaare wurde die durchschnittliche stündliche Abbaurate (∆ AAK/∆ t) berechnet. Wertepaare, bei denen sich Hinweise für eine nichtabgeschlossene Resorptionsphase (z. B. AAK2 > AAK1) ergaben, wurden bei der weiteren Auswertung nicht berücksichtigt. Nach Ermittlung und Elimination von Ausreißern mit Alkoholabbauraten > 0,120 mg/l/h bzw. < 0,030 mg/l/h (MW_alle MNS_ ± 1S) wurden für die Wertepaare mit und ohne Maske (alle, OP, FFP2, Textil) Mittelwerte mit Standardabweichung, Mediane und Interquartilsabstände berechnet. Eine Signifikanzprüfung wurde bei geringer Probandenzahl nicht durchgeführt.

## Ergebnisse

Es wurden 2 weibliche und 4 männliche Probanden zwischen 28 und 37 Jahren in die Studie eingeschlossen. Die aufgenommene Alkoholmenge betrug rechnerisch nach der Widmark-Formel zwischen 122 und 210 ml Gin oder Rum, musste jedoch wegen zu niedriger Ausgangswerte bei allen Probanden ab dem zweiten Untersuchungstag auf 160–260 ml erhöht werden. Die maximale Alkoholbelastung lag dadurch zwischen 0,78 und 0,96 g Ethanol/kgKG und h. Die Ausgangs-AAK-Werte zu Beginn aller Messserien lagen bei 0,149–0,533 mg/l. Der Atemalkoholabbau betrug für alle Teilnehmer durchschnittlich 0,073 mg/l und h (Spannweite 0,041–0,125 mg/l und h), wobei sich die individuellen mittleren Atemalkoholabbauraten der einzelnen Probanden an den 3 Untersuchungstagen um bis zu 0,04 mg/l und h unterschieden. Auf die Darstellung einer über alle Messtage gemittelten individuellen Atemethanoleliminationskurve wurde daher verzichtet.

Bei 3 von 6 Proband*innen verliefen alle 18 Messungen, verteilt auf 3 Untersuchungstage, problem- und fehlerlos. Bei 8 von insgesamt 111 Messungen (7,2 %) wurde durch das Gerät eine Messabweichung dokumentiert, woraufhin die Messung abgebrochen und wiederholt wurde. Dies waren jeweils 4 Messungen mit bzw. ohne MNS. Ursache waren ein Nichterreichen des erforderlichen Atemvolumens in der vorgegebenen Zeit bzw. eine zu starke Differenz der Atemzeiten zwischen den beiden Einzelmessungen. In einem Fall konnte im Zeitfenster für die Atemprobenabgabe kein gültiger Messwert erzielt werden. Bei einem Probanden wurde auf die letzte Messung am 1. Messtag verzichtet, da der AAK-Wert bei der vorangegangenen Messung bereits 0,00 mg/l betrug. Fehlermeldungen, die zumindest theoretisch auf das Tragen der MNS zurückzuführen wären, z. B. unzulässige Abweichungen der Atemtemperaturen zwischen den Einzelmessungen eines Messvorgangs oder Detektion von Mundrestalkohol, wurden nicht beobachtet.

### Wertepaare ohne Maske

Es konnten insgesamt 36 gültige Wertepaare gebildet werden, bei denen zwischenzeitlich keine Maske getragen worden war. In dieser Gruppe wurden 2 Ausreißer beobachtet. Der durchschnittliche Abbau betrug 0,070 ± 0,020 mg/l und h.

### Wertepaare mit Maske

Für alle Zeiträume, in denen eine Maske getragen worden war, konnten 51 gültige AAK-Wertepaare gebildet werden. Die durchschnittliche Abbaurate betrug unbereinigt für alle untersuchten Maskentypen 0,076 ± 0,041 mg/l und h. Nach Bereinigung von 13 Ausreißern (7 Werte > 0,120 mg/l und h, 6 Werte < 0,030 mg/l und h) betrug die mittlere Abbaurate 0,069 ± 0,022 mg/l und h.

Die Wertepaare mit den ausreißerbereinigten AAK-Abbauraten mit und ohne Masken sind in Tab. 3 dargestellt.

**Table 3:** 

Proband	Messwertpaare	Maske	AAK1 (mg/l)	AAK2 (mg/l)	Abbaurate (mg/l und h)	Proband*innen	Messwertpaare	Maske	AAK1 (mg/l)	AAK2 (mg/l)	Abbaurate (mg/l und h)
A	2/3	Ohne	0,129	0,100	0,071	A	3/4	OP	0,100	0,068	0,069
A	4/5	Ohne	0,068	0,035	0,065	B	1/2	OP	0,356	0,314	0,065
A	8/9	Ohne	0,368	0,337	0,053	B	3/4	OP	0,288	0,255	0,064
A	10/11	Ohne	0,306	0,261	0,071	B	5/6	OP	0,230	0,207	0,050
A	14/15	Ohne	0,394	0,342	0,101	C	1/2	OP	0,204	0,183	0,037
A	16/17	Ohne	0,295	0,255	0,086	C	3/4	OP	0,160	0,115	0,089
B	2/3	Ohne	0,314	0,288	0,068	C	5/6	OP	0,075	0,043	0,056
B	4/5	Ohne	0,255	0,230	0,054	D	3/4	OP	0,312	0,290	0,057
B	8/9	Ohne	0,384	0,344	0,075	D	5/6	OP	0,267	0,243	0,050
B	10/11	Ohne	0,309	0,273	0,056	E	1/2	OP	0,473	0,422	0,101
B	14/15	Ohne	0,357	0,315	0,078	E	3/4	OP	0,375	0,339	0,080
B	16/17	Ohne	0,288	0,259	0,056	E	5/6	OP	0,317	0,274	0,089
C	2/3	Ohne	0,183	0,160	0,053	F	3/4	OP	0,324	0,293	0,072
C	4/5	Ohne	0,115	0,075	0,084	F	5/6	OP	0,257	0,228	0,060
C	8/9	Ohne	0,336	0,318	0,044	A	9/10	FFP2	0,337	0,306	0,069
C	14/15	Ohne	0,337	0,309	0,054	B	9/10	FFP2	0,344	0,309	0,078
C	16/17	Ohne	0,257	0,203	0,111	B	11/12	FFP2	0,273	0,262	0,031
D	2/3	Ohne	0,365	0,312	0,099	C	9/10	FFP2	0,318	0,261	0,119
D	4/5	Ohne	0,290	0,267	0,044	C	11/12	FFP2	0,211	0,167	0,100
D	8/9	Ohne	0,350	0,305	0,089	D	9/10	FFP2	0,305	0,282	0,045
D	10/11	Ohne	0,282	0,258	0,047	D	11/12	FFP2	0,258	0,244	0,031
D	14/15	Ohne	0,392	0,337	0,096	E	9/10	FFP2	0,393	0,344	0,102
D	16/17	Ohne	0,299	0,274	0,051	E	11/12	FFP2	0,316	0,285	0,057
E	2/3	Ohne	0,422	0,375	0,092	F	9/10	FFP2	0,323	0,274	0,095
E	4/5	Ohne	0,339	0,317	0,044	F	11/12	FFP2	0,255	0,219	0,057
E	8/9	Ohne	0,441	0,393	0,092	A	13/14	Textil	0,426	0,394	0,067
E	10/11	Ohne	0,344	0,316	0,055	A	15/16	Textil	0,342	0,295	0,093
E	14/16	Ohne	0,416	0,332	0,088	A	17/18	Textil	0,255	0,217	0,058
E	16/17	Ohne	0,332	0,293	0,080	B	15/16	Textil	0,315	0,288	0,058
F	2/3	Ohne	0,375	0,324	0,100	B	17/18	Textil	0,259	0,232	0,048
F	4/5	Ohne	0,293	0,257	0,065	C	13/14	Textil	0,353	0,337	0,033
F	8/9	Ohne	0,361	0,323	0,073	C	15/16	Textil	0,309	0,257	0,108
F	10/11	Ohne	0,274	0,255	0,041	C	17/18	Textil	0,203	0,165	0,099
F	16/17	Ohne	0,270	0,243	0,056	D	15/16	Textil	0,337	0,299	0,080
–						D	17/18	Textil	0,274	0,242	0,066
						E	13/14	Textil	0,453	0,416	0,075
						E	17/18	Textil	0,293	0,257	0,072
						F	15/16	Textil	0,295	0,270	0,052

Tab. 4 gibt eine Übersicht über die mittleren AAK-Abbauraten (MW ± SD) mit und ohne MNS.

**Table 4:** 

	Anzahl, Wertepaare	AAK-Abbaurate (mg/l und h)	SD
Ohne Maske	34	0,070	±0,020
Alle Masken	38	0,069	±0,022
OP	14	0,067	±0,018
FFP2	11	0,071	±0,030
Textil	13	0,070	±0,021

*SD* Standardabweichung

**Figure 3:**
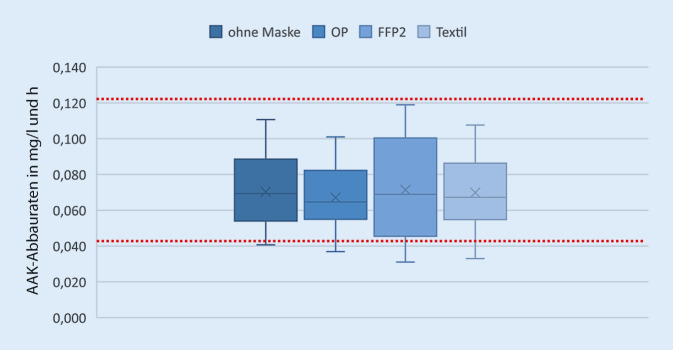

Die Box-Whisker-Plots der AAK-Abbauwerte mit und ohne Masken sind in Abb. 3 dargestellt.

## Diskussion

Seit 1998 ist die Messung der Atemalkoholkonzentration bei Ordnungswidrigkeitsverfahren in Deutschland als Beweismittel zugelassen. Insbesondere in den nachfolgenden Jahren wurde eine Reihe von Einlassungen vorgebracht, in denen eine Verfälschung des Messergebnisses aufgrund verschiedenster Einflussfaktoren geltend gemacht wurde. Dazu zählen z. B. ethanolhaltige Inhalationssprays, Medikamente, Mundspüllösungen und Mundgele [4, 8, 9]. In den letzten Jahren wurde diese Aufstellung durch weitere mögliche Einflussfaktoren wie z. B. ethanolhaltige Zahnprothesenhaftcreme und Zahnpflegemittel [7, 15, 17], alkoholfreie Kaugummis, Pastillen und Zigaretten [14] sowie das Dampfen von E‑Zigaretten [12] ergänzt. Nunmehr bieten die derzeitigen Pandemieumstände durch das Tragen einer MNS eine zusätzliche Variante einer möglichen Beeinflussung.

Da sich die Rekrutierung von Probanden für die Überprüfung dieser Einlassung unter Pandemiebedingungen sowie den daraus resultierenden Vorgaben der Ethikkommission außerordentlich problematisch gestaltete und zudem die Untersuchungsbedingungen coronakonform gestaltet werden mussten, war lediglich eine kleinere Übersichtsstudie möglich. Aufgrund der dadurch nur geringen Probandenzahl war eine statistische Signifikanzprüfung nicht möglich. Dennoch ist aus den Ergebnissen abzuleiten, dass die Atemalkoholabbauraten mit und ohne Maske keine wesentlichen Unterschiede zeigen, lediglich die Spannweite ist bei den AAK-Abbauraten für alle Masken zusammen etwas größer. Systematische Untersuchungen der Atemalkoholabbauraten aus den letzten 2 Jahrzehnten zeigten durchschnittliche AAK-Abbauraten von 0,064–0,080 mg/l und h (Tab. 5). Unter Berücksichtigung der dort angegebenen Standardabweichungen ergaben sich ein oberer Grenzwert von 0,122 mg/l und h [13] und ein unterer Grenzwert von 0,042 mg/l und h [14] für den von außen unbeeinflussten Atemalkoholabbau. Die mittleren 50 % sowie die oberen 25 % der aktuellen Untersuchungsreihe befanden sich sowohl für die gemessenen AAK-Abbauraten ohne Maske als auch für jede einzelne Maske innerhalb des Messbereichs früherer Studien (Abb. 3). Die unteren 25 % aller gemessenen Werte lagen für alle Untersuchungsmodalitäten teilweise nur geringfügig außerhalb des Vergleichsbereiches (0,001–0,011 mg/l und h). Hinweise für eine systematische Überhöhung der Messwerte lassen sich anhand der vorliegenden Untersuchungen im Vergleich mit früheren Untersuchungen ohne Masken nicht erkennen.

**Table 5:** 

Studie	Fragestellung	Analysegerät	*n*	Mittlere AAK-Abbauraten mit SD (in mg/l und h)
Jones et al. 2003 [11]	AAK-Abbauraten	Intoxilyzer 5000S	19	0,080 ± 0,0105^a^
Jachau et al. 2004 [10]	Ethanolelimination in Blut und Urin	Alcotest 7110 MK III Evidential	56	0,079 ± 0,0195
Pavlic et al. 2007 [13]	Abbaurate AAK	Alcotest 7110 MK III Evidential	59	**0,082** **±** **0,04**
Pietsch et al. 2012 [14]	Einfluss von Kaugummis/Pastillen Zigarettenrauch	Alcotest 7110 MK III Evidential	20	**0,064** **±** **0,022**
Sadler et al. 2015 [16]	AAK-Abbauraten	Camic Datamaster Breath Analyser System	18	♂ 0,074 (0,060–0,086) ♀ 0,069 (0,057–0,080)

^a^Wert umgerechnet aus mg/2 l/h; fett gedruckt: obere und untere Grenzen der AAK-Vergleichsabbauraten

Mund-Nase-Schutzmasken sollen die Ausbreitung potenziell virulenter Aerosole in die Umgebungsluft reduzieren, während gasförmige Substanzen weiterhin ungehindert durch das luftdurchlässige Maskengewebe sowie die nichtvermeidbaren Randleckagen strömen [19, 22]. Oral aufgenommenes Ethanol wird zu einem geringen Anteil über die Atmung abgebaut und liegt demnach in der Ausatemluft gasförmig vor. Je nach Maskentyp und anatomischen Gegebenheiten ist laut Literatur von einem Luftreservoir hinter der Maske von ca. 20 ml auszugehen [22]. Nach eigenen orientierenden Volumenmessungen (im Rahmen eines einmaligen Selbstversuches durch Auslitern des Maskentotraumes mit Styroporkügelchen während des Masketragens) lag das Luftreservoir bei ca. 55 ml für OP-Masken und bei ca. 95 ml für FFP2-Masken. Dennoch ist das durch die Maske erweiterte Totraumvolumen im Vergleich zu dem bei der Analyse abgegebenen Gesamtatemvolumen von mehreren Litern vernachlässigbar klein. Durch das notwendige Mindestvolumen von 1,2–3 l bei der Atemprobenabgabe ist daher gewährleistet, dass nicht das obere Totraumvolumen aus der Mundhöhle und den oberen Luftwegen, sondern v. a. die tiefe Lungenluft messtechnisch erfasst wird. Beim Tragen einer MNS wird der Totraum nur um höchstens 0,2 % des geforderten Gesamtvolumens der Atemprobe (in der aktuellen Studie bei max. 4 ± 1,2 l) erweitert. Fehlermeldungen des Messgerätes durch unzulässig hohe Unterschiede zwischen den beiden Einzelmesswerten, wobei die Maske zwischen der ersten und zweiten Messung nicht wieder aufgesetzt wurde, traten während der Messreihen nicht auf.

Die Atemalkoholabbauraten mit und ohne Masken zeigen im direkten Vergleich keine wesentlichen Unterschiede im Median und in der Spannweite (Abb. 3). In der unter den gegenwärtigen Pandemiebedingungen nur in kleinem Umfang rekrutierbaren Studienpopulation waren die Ethanolabbauraten bei OP-Masken und Textilmasken annähernd gleich. Bei den zeitweise in mehreren Bereichen sogar verpflichtend zu tragenden FFP2-Masken (z. B. öffentlicher Nahverkehr in einigen Bundesländern) zeigte sich im Mittel eine höhere stündliche Abbaurate mit einer breiten Streuung, wobei die höchsten Eliminationsraten dennoch innerhalb des Messbereiches früherer Studien ohne Tragen einer MNS lagen (Tab. 5).

## Limitationen

Da die Studie unter Pandemiebedingungen mit einer nur geringen Probandenzahl durchgeführt werden konnte und dadurch keine Signifikanzprüfung möglich war, sind die Ergebnisse nur begrenzt aussagekräftig. Zur Überprüfung wäre eine Testreihe mit größerer Teilnehmerzahl erforderlich.

Der Untersuchungsaufbau erfolgte unter der Maßgabe möglichst reale Messbedingungen zu schaffen, wie sie auch in praxi anzutreffen sind. Die initial berechnete Menge Alkohol, die bei den Proband*innen zu einer BAK von ca. 1 ‰ hätte führen sollen, erwies sich jedoch als nicht ausreichend, um am Ende des Untersuchungstages noch suffiziente Messwerte zu erhalten, sodass die Ethanolmenge um ca. 25 % erhöht werden musste. Die Alkoholbelastung der Teilnehmer lag dadurch mit durchschnittlich 0,88 g Ethanol/kgKG und h im Bereich forcierten Trinkens, wodurch eine Resorptionszeit bis zu 120 min hätte berücksichtigt werden müssen, was jedoch für den zeitlichen Versuchsablauf nicht umsetzbar war und auch nicht den Realbedingungen im polizeilichen Alltag mit meist unbekanntem exakten Trinkende nahekommt.

## Fazit für die Praxis

- Das Tragen einer Mund-Nase-Bedeckung vor der Atemalkoholmessung mit dem Atemalkoholanalysegerät Dräger Alcotest 9510 DE führt weder zur Fehlermeldung noch zum Abbruch der Messung.
- Die Studienresultate sprechen gegen eine systematische Verfälschung der AAK-Messwerte durch das Tragen einer Mund-Nase-Schutzmaske vor der Atemalkoholmessung.
